# Supplementary material for: Does daily activity overlap of seven mesocarnivores vary based on human development?
Source: PLoS One. 2024 Jan 11;19(1):e0288477. doi: 10.1371/journal.pone.0288477 (PMC10783707; doi:10.1371/journal.pone.0288477)
Supplement: S1 Table — (DOCX) [file pone.0288477.s001.docx]

**Supplemental Table 1. Habitat at each of the Snapshot USA camera sites used in analyses.**

| Habitat Type | Number of Sites |
| --- | --- |
| Anthropogenic | 15 |
| Desert | 6 |
| Forest | 145 |
| Grassland | 42 |
| Riparian | 1 |
| Wetlands | 1 |

Habitat classifications for 210 Snapshot USA 2019 and 2020 camera trap locations. Sites were chosen to include mammalian carnivores on an urban to rural gradient.
