# Supplementary material for: Does daily activity overlap of seven mesocarnivores vary based on human development?
Source: PLoS One. 2024 Jan 11;19(1):e0288477. doi: 10.1371/journal.pone.0288477 (PMC10783707; doi:10.1371/journal.pone.0288477)

PLOS ONE

Does daily activity overlap of three mesocarnivores vary based on human development?

--Manuscript Draft--

**Manuscript Number:**	PONE-D-22-35736
**Article Type:**	Research Article
**Full Title:**	Does daily activity overlap of three mesocarnivores vary based on human development?
**Short Title:**	Temporal overlap of mesocarnivores based on human development.
**Corresponding Author:**	University of Arkansas USGS cooperative research unit<br>University of Arkansas Fayetteville<br>Fayetteville, AR UNITED STATES
**Keywords:**	Activity Overlap; Activity Patterns; Coyote; Landscape; Mesocarnivore; Raccoon; Temporal Partitioning; Virginia Opossum
**Abstract:**	Predation and competition can influence the behavior and activity patterns of co-occurring species. Often subordinate species will alter their activity patterns to avoid being active at the same time as larger, dominant species. Human development can complicate interspecies interactions, as not all wildlife responds to human activity in the same manner. While some species may alter their activity to avoid being active when humans are, others may be unaffected or may benefit from being active at the same time as humans to reduce predation risk or competition. To further explore this phenomenon, we used data from a coordinated national camera-trapping program (Snapshot USA) to explore the temporal activity overlap of a large, dominant mesocarnivore, the coyote (Canis latrans) with two subordinate species, Virginia opossum (Didelphis virginiana) and Northern raccoon (Procyon lotor) along a gradient of human development. We found all three species to be predominantly nocturnal and while the subordinate species showed modest changes to their activity patterns in response to increasing human development, the dominant coyote did not alter activity patterns in response to surrounding development. As a result, temporal activity overlap was generally high between all species regardless of development. It appears that competitive and predatory pressures between these three generalist species were insufficient to cause any of them to strongly alter their activity patterns.
**Order of Authors:**	Leah E. McTigue<br>Ellery V Lassiter, PhD<br>Mike Shaw<br>Emily Johansson<br>Ken Wilson<br>Brett A. DeGregorio, PhD, MSc
**Additional Information:**	
**Question**	**Response**
**Financial Disclosure**<br><br>Enter a financial disclosure statement that describes the sources of funding for the work included in this submission. Review the submission guidelines for detailed requirements. View published research articles from *PLOS ONE* for specific examples.	The authors received no specific funding for this work.

Powered by Editorial Manager® and ProduXion Manager® from Aries Systems Corporation

This statement is required for submission and **will appear in the published article** if the submission is accepted. Please make sure it is accurate.<br><br>**Unfunded studies**<br>Enter: *The author(s) received no specific funding for this work.*<br><br>**Funded studies**<br>Enter a statement with the following details:<br>• Initials of the authors who received each award<br>• Grant numbers awarded to each author<br>• The full name of each funder<br>• URL of each funder website<br>• Did the sponsors or funders play any role in the study design, data collection and analysis, decision to publish, or preparation of the manuscript?<br>• **NO** - Include this sentence at the end of your statement: *The funders had no role in study design, data collection and analysis, decision to publish, or preparation of the manuscript.*<br>• **YES** - Specify the role(s) played.<br><br>* typeset	
**Competing Interests**<br><br>Use the instructions below to enter a competing interest statement for this submission. On behalf of all authors, disclose any competing interests that could be perceived to bias this work—acknowledging all financial support and any other relevant financial or non-financial competing interests.<br><br>This statement is required for submission and **will appear in the published article** if the submission is accepted. Please make sure it is accurate and that any funding sources listed in your Funding Information later in the submission form are also declared in your Financial Disclosure statement.<br><br>View published research articles from *PLOS ONE* for specific examples.	The authors have declared that no competing interests exist.

**NO authors have competing interests**<br><br>Enter: *The authors have declared that no competing interests exist.*<br><br>**Authors with competing interests**<br><br>Enter competing interest details beginning with this statement:<br><br>*I have read the journal's policy and the authors of this manuscript have the following competing interests: [insert competing interests here]*<br><br>* typeset	
**Ethics Statement**<br><br>Enter an ethics statement for this submission. This statement is required if the study involved:<br><br>• Human participants<br>• Human specimens or tissue<br>• Vertebrate animals or cephalopods<br>• Vertebrate embryos or tissues<br>• Field research<br><br>Write "N/A" if the submission does not require an ethics statement.<br><br>General guidance is provided below. Consult the submission guidelines for detailed instructions. **Make sure that all information entered here is included in the Methods section of the manuscript.**	NA

**Format for specific study types**<br><br>**Human Subject Research (involving human participants and/or tissue)**<br>• Give the name of the institutional review board or ethics committee that approved the study<br>• Include the approval number and/or a statement indicating approval of this research<br>• Indicate the form of consent obtained (written/oral) or the reason that consent was not obtained (e.g. the data were analyzed anonymously)<br><br>**Animal Research (involving vertebrate animals, embryos or tissues)**<br>• Provide the name of the Institutional Animal Care and Use Committee (IACUC) or other relevant ethics board that reviewed the study protocol, and indicate whether they approved this research or granted a formal waiver of ethical approval<br>• Include an approval number if one was obtained<br>• If the study involved *non-human primates*, add *additional details* about animal welfare and steps taken to ameliorate suffering<br>• If anesthesia, euthanasia, or any kind of animal sacrifice is part of the study, include briefly which substances and/or methods were applied<br><br>**Field Research**<br><br>Include the following details if this study involves the collection of plant, animal, or other materials from a natural setting:<br>• Field permit number<br>• Name of the institution or relevant body that granted permission	
**Data Availability**<br><br>Authors are required to make all data underlying the findings described fully available, without restriction, and from the time of publication. PLOS allows rare exceptions to address legal and ethical concerns. See the PLOS Data Policy and FAQ for detailed information.	Yes - all data are fully available without restriction

A Data Availability Statement describing where the data can be found is required at submission. Your answers to this question constitute the Data Availability Statement and **will be published in the article**, if accepted.<br><br>**Important:** Stating 'data available on request from the author' is not sufficient. If your data are only available upon request, select 'No' for the first question and explain your exceptional situation in the text box.<br><br>Do the authors confirm that all data underlying the findings described in their manuscript are fully available without restriction?	
**Describe where the data may be found in full sentences. If you are copying our sample text, replace any instances of XXX with the appropriate details.**<br><br>• If the data are **held or will be held in a public repository**, include URLs, accession numbers or DOIs. If this information will only be available after acceptance, indicate this by ticking the box below. For example: *All XXX files are available from the XXX database (accession number(s) XXX, XXX.).*<br>• If the data are all contained **within the manuscript and/or Supporting Information files**, enter the following: *All relevant data are within the manuscript and its Supporting Information files.*<br>• If neither of these applies but you are able to provide **details of access elsewhere**, with or without limitations, please do so. For example:<br><br>*Data cannot be shared publicly because of [XXX]. Data are available from the XXX Institutional Data Access / Ethics Committee (contact via XXX) for researchers who meet the criteria for access to confidential data.*<br><br>*The data underlying the results presented in the study are available from (include the name of the third party*	All Data is publicly available through Snapshot USA.<br><br>Publications of data used are as follows:<br>Cove MV, Kays R, Bontrager H, Bresnan C, Lasky M, Frerichs T, Klann R, Lee Jr TE, Crockett SC, Crupi AP, Weiss KC. SNAPSHOT USA 2019: a coordinated national camera trap survey of the United States. 2021 Apr 01; 102(6):e03353<br><br>Kays R, Cove MV, Diaz J, Todd K, Bresnan C, Snider M, Lee Jr TE, Jasper JG, Douglas B, Crupi AP, Weiss KC. SNAPSHOT USA 2020: A second coordinated national camera trap survey of the United States during the COVID-19 pandemic. Ecology. 2022 Jun 05; 103(10):e3775.

and contact information or URL).<br>• This text is appropriate if the data are owned by a third party and authors do not have permission to share the data.<br><br>* typeset	
Additional data availability information:	

Powered by Editorial Manager® and ProduXion Manager® from Aries Systems Corporation

Manuscript

Click here to access/download;Manuscript;McTigue et al._Activity Patterns_Final.docx

Does daily activity overlap of three mesocarnivores vary based on human development?

Temporal overlap of mesocarnivores based on human development.

Leah E. McTigue[*,1], Ellery V. Lassiter[1], Mike Shaw[1], Emily Johansson[1], Ken Wilson[1], and Brett A. DeGregorio[2]

[1]University of Arkansas, Department of Biological Sciences, 850 W. Dickson St. Fayetteville, AR 72701. lmctigue@uark.edu, evlassit@uark.edu, ms161@uark.edu, epjohans@uark.edu, kw101@uark.edu

[2]U.S. Geological Survey. Arkansas Cooperative Fish and Wildlife Research Unit. 850 W. Dickson St. Fayetteville, AR 72701. bdegrego@uark.edu

This draft manuscript is distributed solely for purposes of scientific peer review. Its content is deliberative and predecisional, so it must not be disclosed or released by reviewers. Because the manuscript has not yet been approved for publication by the U.S. Geological Survey (USGS), it does not represent any official USGS finding or policy.

Abstract

Predation and competition can influence the behavior and activity patterns of co-occurring species. Often subordinate species will alter their activity patterns to avoid being active at the same time as larger, dominant species. Human development can complicate interspecies interactions, as not all wildlife responds to human activity in the same manner. While some species may alter their activity to avoid being active when humans are, others may be unaffected or may benefit from being active at the same time as humans to reduce predation risk or competition. To further explore this phenomenon, we used data from a coordinated national camera-trapping program (Snapshot USA) to explore the temporal activity overlap of a large, dominant mesocarnivore, the coyote (*Canis latrans*) with two subordinate species, Virginia opossum (*Didelphis virginiana*) and Northern raccoon (*Procyon lotor*) along a gradient of human development. We found all three species to be predominantly nocturnal and while the subordinate species showed modest changes to their activity patterns in response to increasing human development, the dominant coyote did not alter activity patterns in response to surrounding development. As a result, temporal activity overlap was generally high between all species regardless of development. It appears that competitive and predatory pressures between these three generalist species were insufficient to cause any of them to strongly alter their activity patterns.

Keywords**:** Activity Overlap, Activity Patterns, Coyote, Landscape, Mesocarnivore, Raccoon, Temporal Partitioning, Virginia Opossum

Introduction

Interspecific interactions are key drivers of the structure of wildlife communities with both predation and competition influencing the distribution, behavior, activity patterns, and resource use of co-occurring species (Fischer et al. 2012; Monterroso et al. 2014). Because many species of mammal are capable of being active during the day or night (Grinder and Krausman 2001; Gaynor et al. 2018), they may shift their behavior to maximize access to resources and avoid competitors or predators (Arias-Del Razo et al. 2011). Both predation (or the threat of predation) and competition can cause shifts in behavior such that subordinate or prey species may choose to be active when dominant species are least active (Vanek et al. 2021). Conversely, predators should seek to align their activity patterns with those of their prey to optimize energy gain (Lang, Mann and Farnie 2019). From a competitive standpoint, smaller-bodied or subordinate competitors may be active during times when larger or more dominant competitors are least active to avoid agonistic interactions (Arias-Del Razo et al. 2011). Explorations of activity overlap have provided important insights into how wildlife species behaviorally respond to their environment, predators, and competitors (Gehring and Swihart 2003; Arias-Del Razo et al. 2011; Carver et al. 2011). However, the activity patterns of wildlife are driven not only by co-occurring species but may be strongly influenced by anthropogenic disturbances (Frid and Dill 2002; Gaynor et al. 2018).

Many species of wildlife have adapted to co-occur with humans in developed areas (i.e. urban adapted species; Gallo et al. 2017) and in these areas their temporal activity patterns may differ from more natural settings (Frid and Dill 2002; Gese et al. 2012; Lowery et al. 2012; Cove et al. 2017). When co-occurring with humans, many species respond by becoming active when humans are least active, typically at night (Gehrt et al. 2007; Wang et al. 2015; Gaynor et al.

2018; Moll et al. 2018). This may be particularly true for large predatory species that are often persecuted by humans (Ordeñana et al. 2010; Wang et al. 2015; Smith et al. 2018) and thus sensitive to human activity and development. If co-occurring species respond similarly to humans by shifting to a more nocturnal existence, then the activity overlap between predators and their prey or between competitor species may necessarily be high (Moll et al. 2018). However, not all urban-adapted wildlife species respond to human presence in the same manner even within taxa groups such as mesocarnivores (medium-sized mammalian predators). Thus, species that are more tolerant of human activity may not switch to a nocturnal existence; thereby, reducing interactions with predators or competitors that do so. Furthermore, changes in activity patterns may vary based on the intensity of human development such that changes in activity patterns may vary along a gradient of development (Gese et al. 2012; Cove et al. 2017).

In human-dominated landscapes where larger bodied predator activity may be constrained temporally by an avoidance of humans, subordinate species may alter their activity patterns to avoid overlap, a phenomenon referred to as “development-mediated temporal avoidance” (Atickem et al. 2014; Moll et al. 2018; Breck et al. 2019). In situations where the overlap in activity of predator and prey are reduced or altered due to the presence of humans or development, prey may be shielded by development (Moll et al. 2018; Gámez and Harris 2021). Similarly, interference competition by larger bodied or dominator competitor species may be reduced in human developed areas (Dorresteijn et al. 2015; Shores et al. 2018). Mesocarnivore communities offer an excellent opportunity to study these interactions, because they have adapted to co-occur in human dominated landscapes and are generally well-studied (Magle et al. 2016; Bozek et al. 2017; Greenspan et al. 2018). In North America, the coyote (*Canis latrans*) is the largest mesocarnivore in most ecosystems and is considered dominant over many co-occurring,

smaller-bodied mesopredators species, such as northern raccoons (hereafter raccoon: *Procyon lotor*) and Virginia opossum (hereafter, opossum: *Didelphis virginiana*), due to competitive size advantage or predator relationship (Gipson and Kamler 2001). Coyotes, racoons, and opossums overlap in geographic range across much of North America and occupy habitats along a gradient of development (Prange and Gehrt 2004; Rodriguez et al. 2021). Both racoons and opossums are preyed upon by coyote, however, predation risk for opossums is thought to be greater than for racoons (Gipson and Kamler 2001; Gehrt and Clark 2003). Additionally, all three species are primarily nocturnal (Alejandra et al. 2020, Chitwood et al. 2020), though recent research shows that raccoons and opossums may shift activity to crepuscular hours when in proximity to humans (Mims et al. 2022).

Here, our overall objective was to evaluate whether human development mediates the sympatry of a larger, more dominant mesocarnivore (coyote) with two smaller, subordinate mesocarnivores (Virginia opossum and raccoon). Using camera trap data from a nationwide collaborative effort (Snapshot USA; Cove et al. 2021; Kays et al. 2022) our specific objectives were to 1) characterize the activity patterns of the three species at differing levels of human development, 2) evaluate the degree of temporal activity overlap between each species pair, and 3) determine if the degree of surrounding human development correlates with changes in activity overlap between each pair of species. We predicted that all three species would primarily be nocturnal and thus temporal activity overlap would generally be high. However, we predicted that the degree of overlap between coyote and opossum would be lower than between coyote and raccoon, because opossum are more at risk of predation by coyote (Gipson and Kamler 2001). Additionally, we predicted that coyote activity would be most temporally constrained in areas of high human development due to the threat of human persecution (Tigas et al. 2002) and therefore

overlap between coyotes with raccoon and opossum would decrease in heavily developed areas relative to undeveloped or moderately developed areas.

Methods

Data Selection

We used camera trap data from Snapshot USA (Cove et al. 2021; Kays et al. 2022). Snapshot USA is a nationwide coordinated camera trapping project in which contributors deploy motion-triggered infrared cameras annually in a standardized manner between September 1st and October 31st. At each study site, each contributor deploys at least 8 cameras in a single type of habitat (forest, grassland, desert, anthropogenic, etc) and aims to collect a minimum of 400 trap nights. Cameras are placed at least 200m apart, but no more than 5km apart. We only included data from study sites where all three of our focal species co-occurred and were detected.

To characterize how human development surrounding each Snapshot USA site, we created 500m buffers around each camera using ArcGIS (ArcGIS Pro; Esri Inc, Redlands, CA). We chose 500m buffers because this spatial scale has been shown to be relevant to the occurrence and behavior of our focal species in previous studies (Fidino et al. 2016, Magle et al. 2016, Gallo et al. 2017). Within the 500m buffer, we used the Silvis Housing Unit Density layer to calculate the maximum Housing Unit Density (HUD) occurring around each camera as a metric of human development (Hammer et al. 2004). We then averaged this for all cameras at a given site. We designated three categories to group each site: undeveloped (HUD = 0-12 homes/km^2), moderately developed (HUD = 12-100 homes/km^2), or heavily developed (HUD > 100 homes/km^2; Hammer et al. 2004).

Activity Patterns and Overlap

For each detection of a focal species, we extracted the date and time of the detection. We then determined if the detection occurred during the day, night, around dusk, or around dawn by calculating location and date-specific sunset and sunrise times using the R package sunCalc (Thieurmel and Elmarhraoui 2019). Detections occurring within an hour of sunset were categorized as dusk, and detections occurring within an hour of sunrise were categorized as dawn. Nocturnal events (classified as night) occurred after dusk and before dawn. Diurnal events (classified as day) occurred after dawn and before dusk. At each site, we calculated the proportion of events occurring during each of the four daily activity periods (dawn, dusk, day, or night). We compared the proportion of events occurring during each of the four time periods across three development groups (undeveloped, moderately developed, and heavily developed) using a Kruskal-Wallis test. We then used Dunn tests with Bonferroni corrections to make pair-wise comparisons between the development categories. We used non-parametric tests due to non-normality in the data.

To calcula,te the activity overlap for each of the three species pairs, we used the R package overlap (Meredith and Ridout 2021). We first converted the time of each focal species detection to radians to account for the circular nature of time. We then calculated a site and species-specific non-parametric kernel around the detection times to quantify when each species was most active during the diel period (Ridout and Linkie 2009). For each site, we calculated the area under the kernel curve that overlapped between each of the three species pairs, which was quantified using a 0-1 index with 0 indicating no overlap and 1 indicating complete temporal overlap (Schmid and Schmidt 2006). We used Δ_1 for small sample sizes because most sites had fewer than 75 detections per species (Schmid and Schmidt 2006; Ridout and Linkie 2009). We

excluded sites where detections were too few to generate kernel density estimates. We generated confidence intervals around each overlap index from bootstrap samples (1000 iterations). We then compared the amount of overlap between each species pair across the three development groups using Kruskal-Wallis and Dunn tests.

Results

We included data from 82 Snapshot USA 2019 and 2020 sites where all three species (coyote, opossum, and raccoon) were detected. In total, we included 2,633 coyote detections, 13,369 raccoon detections, and 4,639 opossum detections.

All three species were predominantly nocturnal (Fig 1-3). Opossum were the most strongly nocturnal species with the proportion of nocturnal events at each site ranging from 57.14 – 100 (mean ± 1 SD: 90.45 ± 8.8, Fig 1). Raccoons were predominately active at night (78.52 ± 11.4) with some activity around dawn (Fig 2). Similarly, coyote activity was greatest at night (66.78 ± 23.6), but also included low but consistent levels of activity across all other time periods (Fig 3).

Fig 1. **Daily activity patterns for Virginia opossum (*Didelphis virginiana*) along an urban to rural gradient.** The proportion of Virginia opossum (*Didelphis virginiana*) activity occurring during different diel periods based on surrounding levels of development as measured by housing unit density (HUD). Data were collected from 82 study sites across North America as part of Snapshot USA 2019 and 2020 (Cove et al. 2021; Kays et al. 2022).

Fig 2. **Daily activity patterns for northern raccoon (*Procyon lotor*) along an urban to rural gradient.** The proportion of northern Raccoon (*Procyon lotor*) activity occurring during different

diel periods based on surrounding levels of development as measured by housing unit density (HUD). Data were collected from 82 study sites across North America as part of Snapshot USA 2019 and 2020 (Cove et al. 2021; Kays et al. 2022).

Fig 3. Daily activity patterns for coyote (*Canis latrans*) along an urban to rural gradient. The proportion of coyote (*Canis latrans*) activity occurring during different diel periods based on surrounding levels of development as measured by housing unit density (HUD). Derived from data collected across 82 study sites across North America as part of Snapshot USA 2019 and 2020 (Cove et al. 2021; Kays et al. 2022).

Opossums and raccoons shifted timing of activity in response to human development (Fig 1-2). Opossum nocturnal activity (X^2 = 8.479, *df* = 2, *P* = 0.014) and dusk activity (X^2 = 6.024, *df* = 2, *P* = 0.049) differed across levels of human development. Opossum nocturnal activity was significantly greater at undeveloped sites than developed sites (Fig 1). However, at moderately developed sites, opossum activity was greatest at dusk (X^2 = 6.024, *df* = 2, *P* = 0.049). Raccoon nocturnal activity varied by development type (X^2 = 6.75, *df* = 2, *P* = 0.034). However, raccoon nocturnal activity was greater at developed sites than undeveloped (X^2 = 6.75, *df* = 2, *P* = 0.047; Fig 2). Contrary to our prediction, the timing of coyote activity was not affected by human development (Day: X^2 = 1.696, *df* = 2, *P* = .428; Dusk: X^2 = 3.702, *df* = 2, *P* = 0.157; Dawn: X^2 = 0.0583, *df* = 2, *P* = 0.971; Night: X^2 = 2.013, *df* = 2, *P* = 0.366; Fig 3).

In general, overlap in the timing of activity between each species pair was high (Fig 4). The greatest observed overlap was between the subordinate species, opossum and raccoon, ranging from 0.018 to 0.923 (mean ± 1 SD: 0.714 ± 0.158). Coyote and racoon overlap values ranged from 0.087 - 0.904 (0.609 ± 0.189), and were slightly greater than coyote and opossum, for which overlap ranged from 0.01 to 0.907 (0.538 ± 0.213). We found no significant differences

in overlap between the three species pairs based on development (coyote + opossum: X^2 = 1.97, *df* = 2, *P* = 0.373; coyote + raccoon: X^2 = 3.499, *df* = 2, *P* = 0.174; opossum + raccoon: X^2 = 4.718, *df* = 2, *P* = 0.095; Fig 4).

Fig 4. Temporal overlap patterns between pairs of three mesocarnivores along an urban to rural gradient. Daily activity overlap between pairs of mesocarnivores including– coyote (*Canis latrans*), Virginia opossum (*Didelphis virginiana*) and northern raccoon (*Procyon lotor*). We compare mean overlap coefficient and standard error values for each species pair at three levels of development as measured by housing unit density (HUD) (coyote + opossum (CO), coyote + raccoon (CR), and opossum + raccoon (OR)).

Discussion

As predicted, opossum, raccoon, and coyote were generally nocturnal across the 82 sites (Andelt 1985; Grinder and Krausman 2001; Gehrt et al. 2011; Chitwood et al. 2020; Mims 2022) but did show the ability to be active during each of the diel activity periods (Fig 1-3). Both raccoons and opossum showed very little diurnal activity but had small peaks of activity during the crepuscular periods, particularly around dawn (Fig 1-2). Of the three species, coyote showed a propensity to have some level of diurnal activity at most sites, a phenomenon that has been reported previously (McClennen et al. 2001; Chitwood et al. 2020).

Because all three species were primarily nocturnal, we observed relatively high rates of activity overlap between each species pair (Fig 4). We saw the greatest degree of overlap between opossum and raccoon despite the fact that these species compete for food due to their overlapping and opportunistic diets (Kasparian et al. 2004). Evidence of direct competition

between these species is limited but it has been shown that opossum expand their habitat use when raccoons are removed from an area (Ginger et al. 2003). Our data suggest that competition between these two species is not strong enough to cause a temporal shift in the activity of either species.

Coyote and opossum had the least amount of overlap in activity, which supports our initial prediction (Fig 4). Opossum experience high rates of predation by coyotes (up to 76% annual mortality: Gipson and Kamler 2001; Kamler and Gipson 2004) and should benefit by avoiding overlap with coyotes when possible (Wang et al. 2015). We had predicted that this predation pressure would be sufficient to elicit changes in the activity patterns of the opossum to avoid coyote. Despite this pressure, temporal activity overlap between the species was still relatively high (>50%). Opossum do not appear inclined to shift their activity away from primarily being active at night. Opossum are vulnerable to a wide range of predators (including avian predators, mammalian predators, and domestic animals: Crooks and Soule 1999) and this prevents them from changing their activity patterns in response to a single generalist predator, such as the coyote. Given the relatively short lifespan of the typical opossum (McRuer et al. 2009), they may prioritize food and mate acquisition over predator avoidance.

We also report a high degree of activity overlap between coyote and racoons. Coyote have been documented preying upon racoons, although it is generally thought that predation events are infrequent (Gehrt et al. 2003, 2007). Several studies have shown little evidence of racoons spatially avoiding areas due to coyote presence (Gehrt et al. 2007; Fidino et al. 2018) and racoons have even been documented sharing anthropogenic resources (bait stations) with coyotes (Chitwood et al. 2020). Thus, risk of predation from coyote is likely not strong enough to elicit changes in activity of the subordinate racoon. However, in some systems coyotes have been

shown to be responsible for up to 40% of mortality events of raccoons (Kamler and Gipson 2004) so there may be regional variation in predation pressure from coyotes on raccoons.

Contrary to our predictions, we found only modest changes in the activity patterns of our focal species across the three categories of development (Fig 1-3). Past research has reported that some species will avoid conflict with humans by being active at later hours of the night (DeGregorio et al. 2021; Lewis et al. 2021). Our results showed that opossum were more active around dusk when surrounded by moderate development and were most nocturnal at undeveloped sites. Raccoons were most nocturnal in developed areas, potentially as a mechanism to avoid being active with humans while still making use of anthropogenic resources (Prange and Gehrt 2004). These results are in contrast to a recent report that showed opossum in a major city area being more crepuscular and raccoons being exclusively nocturnal compared to their counterparts inhabiting natural areas (Mims et al. 2022). Despite numerous reports of coyote switching to nocturnal activity in the presence of humans (Gehrt et al. 2011; Lowry et al. 2012; Greenspan et al. 2018) we found little evidence of range-wide activity shifts of coyotes in response to development (Fig 3). It has been suggested that as human development expands, larger mesopredators, such as coyotes, are becoming bolder and less restrained in when their activity occurrs (Breck et al. 2019).

Due to the scale and scope of Snapshot USA, there was wide variation in the type of development at study sites, and this may have contributed to our relative lack of evidence for consistent shifts in behavior. The way in which wildlife respond to human development are often context dependent (Prevedello et al. 2013; Coppes et al. 2018) and wildlife responses to development may vary regionally or in response to the characteristics of that specific development. Our broad approach likely missed much of the nuance that would have contributed

to a better understanding of how wildlife responds to their environment. Due to the modest shifts in the activity of our focal species, we failed to detected changes in overlap between the species based on the surrounding human development. Future research would benefit from including focal species with even more plasticity in the timing of their activity as opposed to species such as racoon and opossum that are primarily nocturnal or by focusing on more specialized predator – prey models rather than the generalist species we focused on.

Acknowledgements

Thank you to Roland Kays and the Snapshot USA team for access to data and assistance in refining our research question. Thanks to the University of Arkansas Dept of Biological Sciences for student support and to Arkansas Game and Fish Commission for funding this project under cooperative agreement No. 1434-04HQRU1567. Any use of trade, firm, or product names is for descriptive purposes only and does not imply endorsement by the U.S. Government. Data are available the Smithsonian's eMammal data repository https://emammal.si.edu/analysis/data-download.

Literature Cited

Alejandra BS, Osciel SN, Jess GG. Daily activity patterns and relative abundance of medium and large mammals in a communal natural protected area on the central coast of Oaxaca, Mexico. International Journal of Biodiversity and Conservation. 2020 Jul 31; 12(3):159-68.

Andelt WF. Behavioral ecology of coyotes in south Texas. Wildlife Monographs. 1985 Oct 01:3-45.

Arias-Del Razo I, Hernández L, Laundré JW, Myers O. Do predator and prey foraging activity patterns match? A study of coyotes (Canis latrans), and lagomorphs (Lepus californicus and Sylvilagus audobonii). Journal of Arid Environments. 2011 Feb 01;75(2):112-8.

Atickem A, Loe LE, Stenseth NC. Individual heterogeneity in use of human shields by mountain nyala. Ethology. 2014 Apr 30;120(7):715-25.

Fournier DA, Skaug HJ, Ancheta J, Ianelli J, Magnusson A, Maunder MN, Nielsen A, Sibert J. AD Model Builder: using automatic differentiation for statistical inference of highly parameterized complex nonlinear models. Optimization Methods and Software. 2012 Apr 01;27(2):233-49.

Bozek CK, Prange S, Gehrt SD. The influence of anthropogenic resources on multi-scale habitat selection by raccoons. Urban Ecosystems. 2007 Aug 18;10(4):413-25.

Carver BD, Kennedy ML, Houston AE, Franklin SB. Assessment of temporal partitioning in foraging patterns of syntopic Virginia opossums and raccoons. Journal of Mammalogy. 2011 Feb 16;92(1):134-9.

Coppes J, Nopp-Mayr U, Grünschachner-Berger V, Storch I, Suchant R, Braunisch V. Habitat suitability modulates the response of wildlife to human recreation. Biological conservation. 2018 Nov 01;227:56-64.

Cove MV, Gardner B, Simons TR, Kays R, O'Connell AF. Free-ranging domestic cats (Felis catus) on public lands: estimating density, activity, and diet in the Florida Keys. Biological Invasions. 2017 Aug 05;20(2):333-44.

Cove MV, Kays R, Bontrager H, Bresnan C, Lasky M, Frerichs T, Klann R, Lee Jr TE, Crockett SC, Crupi AP, Weiss KC. SNAPSHOT USA 2019: a coordinated national camera trap survey of the United States. 2021 Apr 01; 102(6):e03353

Chitwood MC, Lashley MA, Higdon SD, DePerno CS, Moorman CE. Raccoon vigilance and activity patterns when sympatric with coyotes. Diversity. 2020 Sep 04;12(9):341.

Crooks KR, Soulé ME. Mesopredator release and avifaunal extinctions in a fragmented system. Nature. 1999 Aug 05;400(6744):563-6.

DeGregorio BA, Gale C, V Lassiter E, Massey A, Roberts CP, T Veon J. Nine- banded armadillo (Dasypus novemcinctus) activity patterns are influenced by human activity. Ecology and Evolution. 2021 Nov 03;11(22):15874-81.

Dorresteijn I, Schultner J, Nimmo DG, Fischer J, Hanspach J, Kuemmerle T, Kehoe L, Ritchie EG. Incorporating anthropogenic effects into trophic ecology: predator–prey interactions in a human-dominated landscape. Proceedings of the Royal Society B: Biological Sciences. 2015 Sep 07;282(1814):20151602.

Fidino MA, Lehrer EW, Magle SB. Habitat dynamics of the Virginia opossum in a highly urban landscape. The American Midland Naturalist. 2016 Apr 01;175(2):155-67.

Fidino M, Simonis JL, Magle SB. A multistate dynamic occupancy model to estimate local colonization–extinction rates and patterns of co- occurrence between two or more interacting species. Methods in Ecology and Evolution. 2018 Oct 30;10(2):233-44.

Fischer JD, Cleeton SH, Lyons TP, Miller JR. Urbanization and the predation paradox: the role of trophic dynamics in structuring vertebrate communities. Bioscience. 2012 Sep 01;62(9):809-18.

Frid A, Dill L. Human-caused disturbance stimuli as a form of predation risk. Conservation ecology. 2002 Jun 01;6(1).

Gámez S, Harris NC. Living in the concrete jungle: carnivore spatial ecology in urban parks. Ecological Applications. 2021 Jun 23;31(6):e02393.

Gallo T, Fidino M, Lehrer EW, Magle SB. Mammal diversity and metacommunity dynamics in urban green spaces: implications for urban wildlife conservation. Ecological Applications. 2017 Aug 27;27(8):2330-41.

Gaynor KM, Hojnowski CE, Carter NH, Brashares JS. The influence of human disturbance on wildlife nocturnality. Science. 2018 Jun 15;360(6394):1232-5.

Gehring TM, Swihart RK. Body size, niche breadth, and ecologically scaled responses to habitat fragmentation: mammalian predators in an agricultural landscape. Biological conservation. 2003 Feb 01;109(2):283-95.

Gehrt SD, Clark WR. Raccoons, coyotes, and reflections on the mesopredator release hypothesis. Wildlife Society Bulletin (1973-2006). 2003 Oct 01;31(3):836-42.

Gehrt SD, Prange S. Interference competition between coyotes and raccoons: a test of the mesopredator release hypothesis. Behavioral Ecology. 2007 Jan 01;18(1):204-14.

Gehrt SD, Brown JL, Anchor C. Is the urban coyote a misanthropic synanthrope? The case from Chicago. Cities and the Environment (CATE). 2011;4(1):3.

Gese EM, Morey PS, Gehrt SD. Influence of the urban matrix on space use of coyotes in the Chicago metropolitan area. Journal of Ethology. 2012 Aug 03;30(3):413-25.

Ginger SM, Hellgren EC, Kasparian MA, Levesque LP, Engle DM, Leslie DM. Niche shift by Virginia opossum following reduction of a putative competitor, the raccoon. Journal of Mammalogy. 2003 Nov 21;84(4):1279-91.

Gipson PS, Kamler JF. Survival and home ranges of opossums in northeastern Kansas. The Southwestern Naturalist. 2001 Jun 01:178-82.

Greenspan E, Nielsen CK, Cassel KW. Potential distribution of coyotes (Canis latrans), Virginia opossums (Didelphis virginiana), striped skunks (Mephitis mephitis), and raccoons (Procyon lotor) in the Chicago Metropolitan Area. Urban Ecosystems. 2018 Jun 28;21(5):983-97.

Grinder MI, Krausman PR. Home range, habitat use, and nocturnal activity of coyotes in an urban environment. The Journal of wildlife management. 2001 Oct 01:887-98.

Hammer RB, Stewart SI, Winkler RL, Radeloff VC, Voss PR. Characterizing dynamic spatial and temporal residential density patterns from 1940–1990 across the North Central United States. Landscape and Urban Planning. 2004 Aug 15;69(2-3):183-99.

Kamler JF, Gipson PS. Survival and cause-specific mortality among furbearers in a protected area. The American midland naturalist. 2004 Jan 01;151(1):27-34.

Kasparian MA, Hellgren EC, Ginger SM, Levesque LP, Clark JE, Winkelman DL, Engle DM. Population characteristics of Virginia opossum in the cross timbers during raccoon reduction. The American midland naturalist. 2004 Jan 01;151(1):154-63.

Kays R, Cove MV, Diaz J, Todd K, Bresnan C, Snider M, Lee Jr TE, Jasper JG, Douglas B, Crupi AP, Weiss KC. SNAPSHOT USA 2020: A second coordinated national camera trap survey of the United States during the COVID- 19 pandemic. Ecology. 2022 Jun 05; 103(10):e3775.

Lewis JS, Spaulding S, Swanson H, Keeley W, Gramza AR, VandeWoude S, Crooks KR. Human activity influences wildlife populations and activity patterns: implications for spatial and temporal refuges. Ecosphere. 2021 May 13;12(5):e03487.

Lowry H, Lill A, Wong BB. Behavioural responses of wildlife to urban environments. Biological reviews. 2012 Dec 24;88(3):537-49.

Magle SB, Lehrer EW, Fidino M. Urban mesopredator distribution: examining the relative effects of landscape and socioeconomic factors. Animal Conservation. 2015 Aug 27;19(2):163-75.

McClennan N, , Wigglesworth RR, Anderson SH, Wachob DG. The effect of suburban and agricultural development on the activity patterns of coyotes (Canis latrans). The American Midland Naturalist. 2001 Jul 01;146(1):27-36.

McRuer DL, Jones KD. Behavioral and nutritional aspects of the Virginian opossum (Didelphis virginiana). Veterinary Clinics of North America: Exotic Animal Practice. 2009 May 01;12(2):217-36.

Meredith M, Ridout MS. The overlap package. Central R Archive Network Online. 2014 Jun 18. Available at: https://kar.kent.ac.uk/41474/

Mims DM, Yasuda SA, Jordan MJ. Contrasting activity times between raccoons (procyon lotor) and virginia opossums (didelphis virginiana) in urban green spaces. Northwestern Naturalist. 2022 Mar 11;103(1):63-75.

Moll RJ, Cepek JD, Lorch PD, Dennis PM, Tans E, Robison T, Millspaugh JJ, Montgomery RA. What does urbanization actually mean? A framework for urban metrics in wildlife research. Journal of Applied Ecology. 2019 Feb 12;56(5):1289-300.

Moll RJ, Cepek JD, Lorch PD, Dennis PM, Robison T, Millspaugh JJ, Montgomery RA. Humans and urban development mediate the sympatry of competing carnivores. Urban Ecosystems. 2018 Apr 17;21(4):765-78.

Monterroso P, Alves PC, Ferreras P. Plasticity in circadian activity patterns of mesocarnivores in Southwestern Europe: implications for species coexistence. Behavioral Ecology and Sociobiology. 2014 Jun 08;68(9):1403-17.

Ordeñana MA, Crooks KR, Boydston EE, Fisher RN, Lyren LM, Siudyla S, Haas CD, Harris S, Hathaway SA, Turschak GM, Miles AK. Effects of urbanization on carnivore species distribution and richness. Journal of Mammalogy. 2010 Dec 16;91(6):1322-31.

Prange S, Gehrt SD. Changes in mesopredator-community structure in response to urbanization. Canadian Journal of Zoology. 2004 Nov 01;82(11):1804-17.

Prevedello JA, Dickman CR, Vieira MV, Vieira EM. Population responses of small mammals to food supply and predators: a global meta- analysis. Journal of Animal Ecology. 2013 Apr 05;82(5):927-36.

Ridout MS, Linkie M. Estimating overlap of daily activity patterns from camera trap data. Journal of Agricultural, Biological, and Environmental Statistics. 2009 Sep;14(3):322-37.

Rodriguez JT, Lesmeister DB, Levi T. Mesocarnivore landscape use along a gradient of urban, rural, and forest cover. PeerJ. 2021 Apr 06;9:e11083.

Schmid F, Schmidt A. Nonparametric estimation of the coefficient of overlapping—theory and empirical application. Computational statistics & data analysis. 2006 Mar 10;50(6):1583-96.

Shores CR, Dellinger JA, Newkirk ES, Kachel SM, Wirsing AJ. Mesopredators change temporal activity in response to a recolonizing apex predator. Behavioral Ecology. 2018 Jan 16;30(5):1324-35.

Smith JA, Thomas AC, Levi T, Wang Y, Wilmers CC. Human activity reduces niche partitioning among three widespread mesocarnivores. Oikos. 2018 Jan 16;127(6):890-901.

Thieurmel B, Elmarhraoui A, Thieurmel MB. Package ‘suncalc’. 2019 Apr 2. Available at: https://cran. r-project. org/web/packages/suncalc/suncalc.pdf.

Tigas LA, Van Vuren DH, Sauvajot RM. Behavioral responses of bobcats and coyotes to habitat fragmentation and corridors in an urban environment. Biological Conservation. 2002 Dec 1;108(3):299-306.

Vanek JP, Rutter AU, Preuss TS, Jones HP, Glowacki GA. Anthropogenic factors influence the occupancy of an invasive carnivore in a suburban preserve system. Urban Ecosystems. 2021 Feb;24(1):113-26.

Wang Y, Allen ML, Wilmers CC. Mesopredator spatial and temporal responses to large predators and human development in the Santa Cruz Mountains of California. Biological Conservation. 2015 Oct 1;190:23-33.

Click here to access/download;Figure;Fig1.tiff

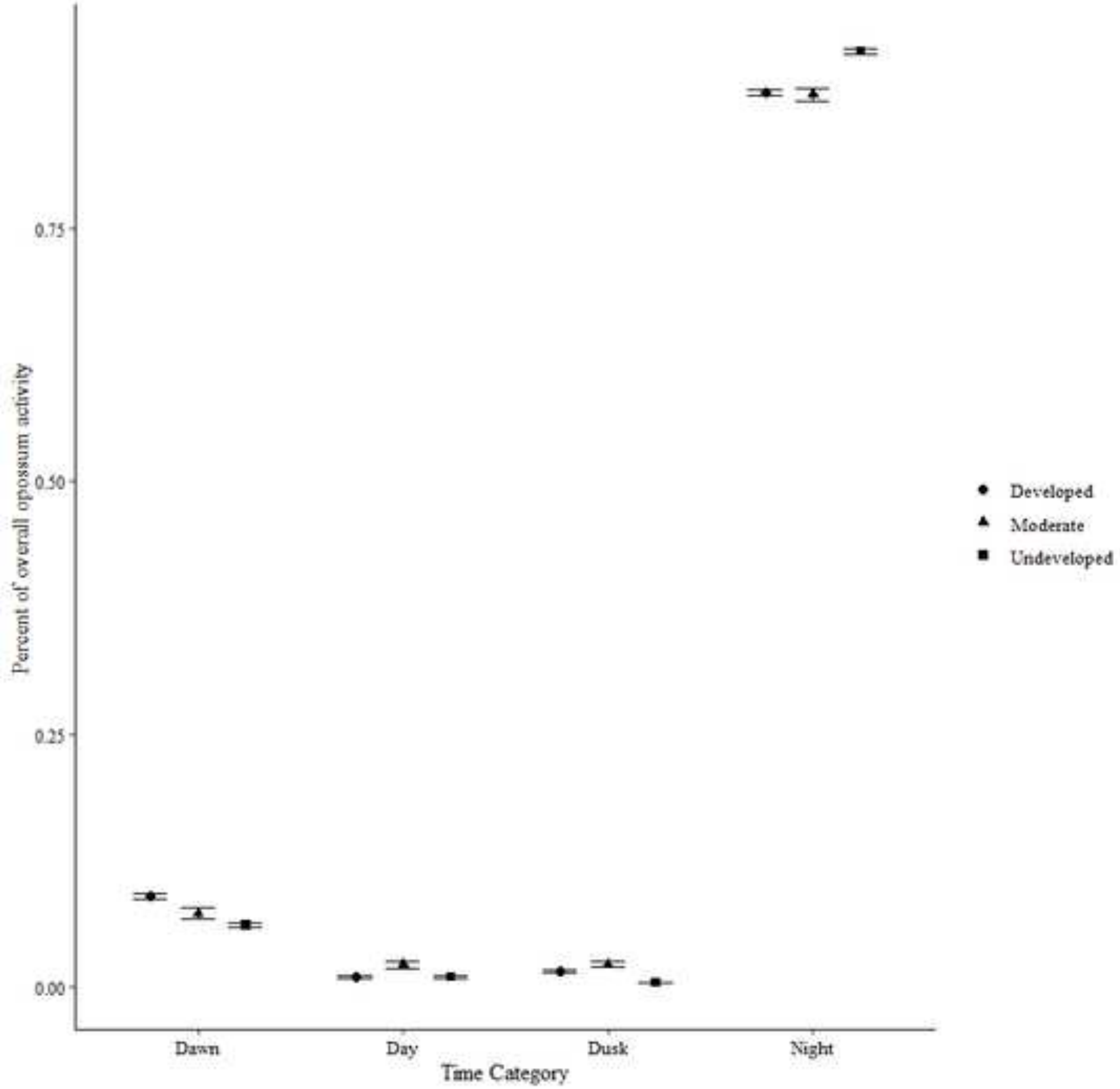

 Click here to access/download;Figure;Fig2.tiff

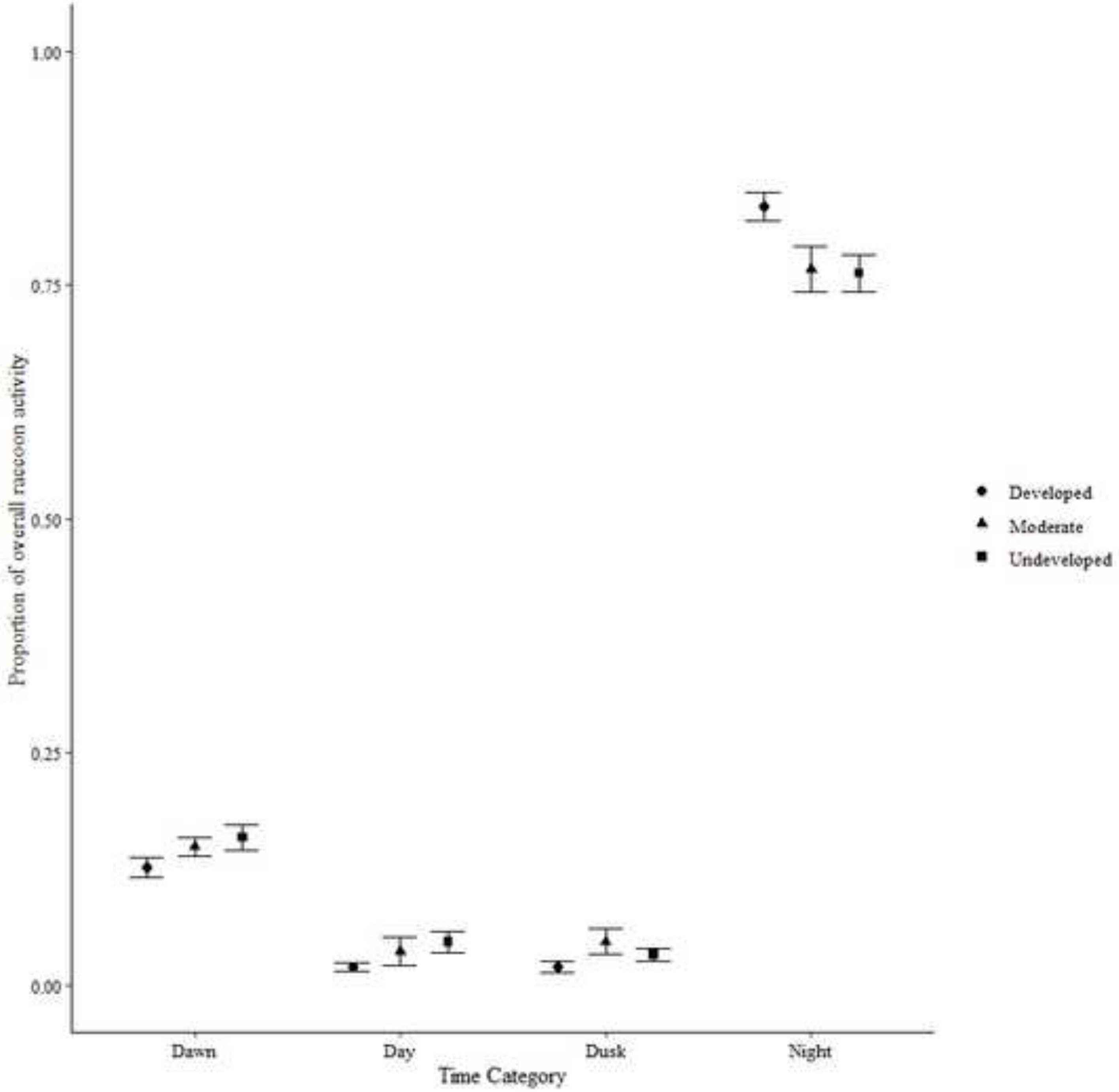

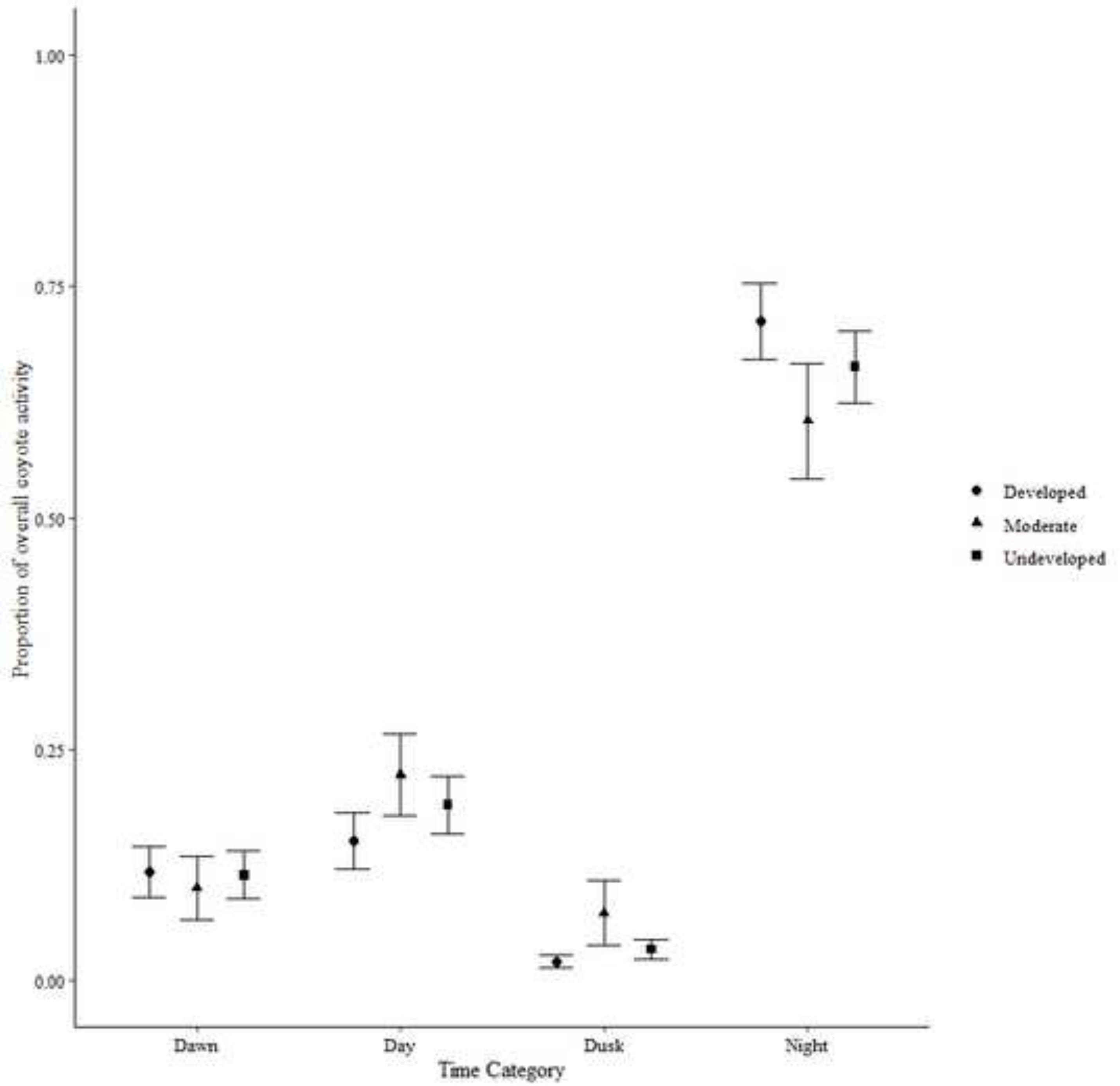
1.00
0.75
0.50
0.25
0.00
Proportion of overall coyote activity
Dawn
Day
Dusk
Night
Time Category
Developed
Moderate
Undeveloped

 Click here to access/download;Figure;Fig4.tiff

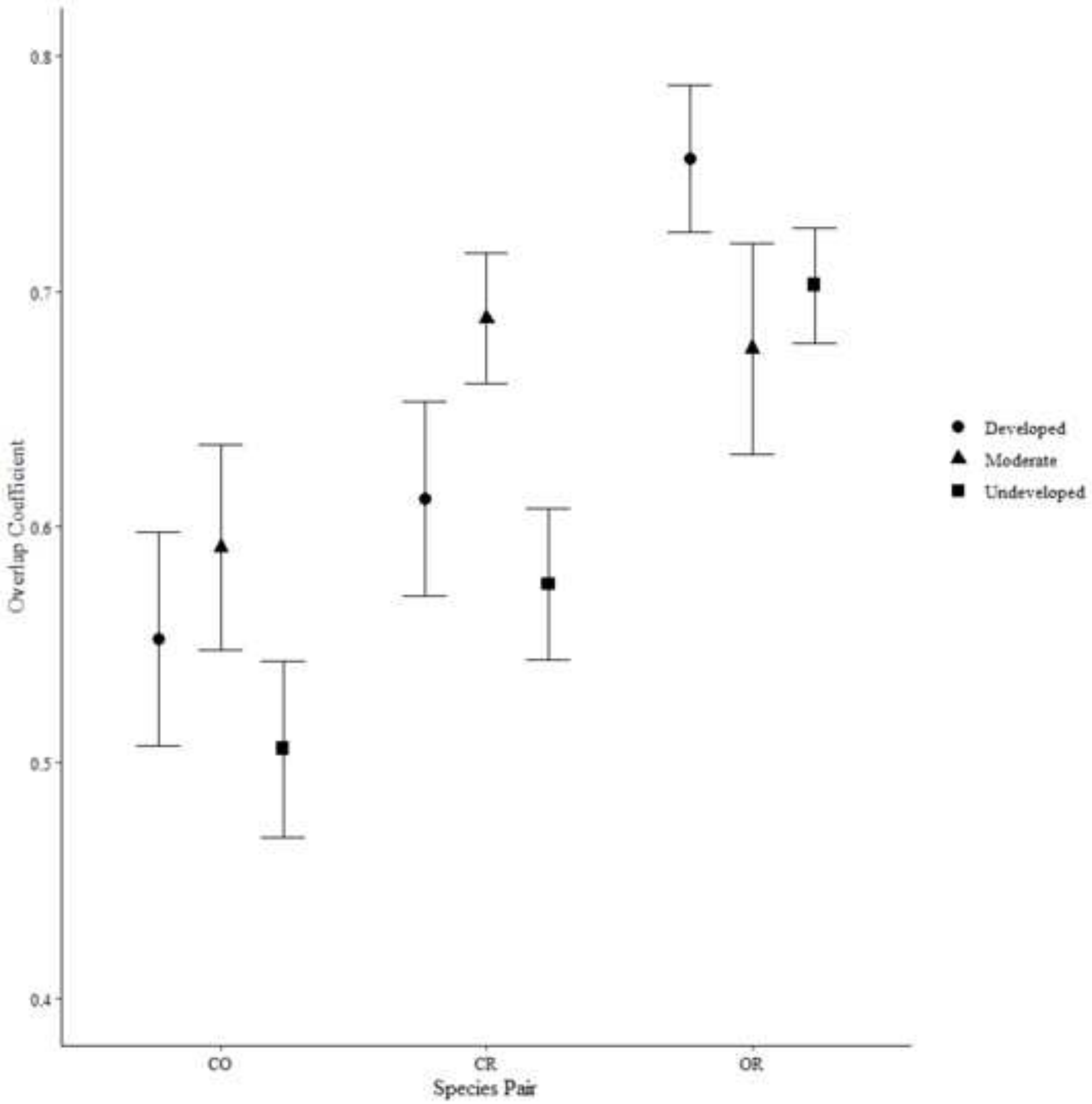

Supplement: Attachment — Submitted filename: PONE-D-22-35736.pdf [file pone.0288477.s003.pdf]
