# Supplementary material for: Does daily activity overlap of seven mesocarnivores vary based on human development?
Source: PLoS One. 2024 Jan 11;19(1):e0288477. doi: 10.1371/journal.pone.0288477 (PMC10783707; doi:10.1371/journal.pone.0288477)
Supplement: Attachment — Submitted filename: Reviewer Response Document_McTigue et al..docx [file pone.0288477.s004.docx]

Additional Editor Comments:

Dear Authors,
I agree with the two reviewers that the MS can be improved. To ensure the Editor and Reviewers will be able to recommend that your revised manuscript is accepted, please pay careful attention to each of the comments that have been pasted underneath this email, and in particular those by Reviewer 1. This way we can avoid future rounds of clarifications and revisions, moving swiftly to a decision.

Reviewers' comments:

Reviewer's Responses to Questions

**Comments to the Author**

**Reviewer #1: PLoS ONE Review:

I thank the editor and authors for the opportunity to review this manuscript. I hope the authors find my review both helpful and constructive. I’ve organized this review by section, where I first give my general comments followed by more-specific line comments.

In this study, the authors investigate whether the temporal overlap of three mesocarnivores (coyote, Virginal opossum, and northern raccoon) varies in relation to human development by utilizing the large Snapshot USA datasets from 2019 and 2020. In general, they find that although species-specific responses to development occur, the majority of activity for all three species remains relatively nocturnal and the resultant overlap between species pairs does not significantly change across development levels. The authors conclude that competitive or predatory pressures between the three species are insufficient in eliciting a behavioral response across a gradient of human development.

In general, I very much enjoyed this manuscript, and the merit for such a study is obvious. The methods and questions were succinct and well-developed, and the paper had a nice flow throughout. However, especially with a dataset as large as Snapshot USA, I feel like the authors could go further, and I urge them to do so in order to publish this work in a higher-impact journal such as this. Specifically, I’m curious why the authors selected only three mesocarnivores from the Snapshot USA dataset. I think more should be included (e.g., bobcat, red fox, grey fox, striped skunk), especially since recent work has shown connections between the species mentioned above and coyotes, in particular (citations provided below). Furthermore, the Snapshot USA dataset includes a decent amount of data on larger apex predators (e.g., wolf, puma, black bear), and I would be extremely curious to include these species in this study as well. Taking a look at how different species assemblages may affect the overlap between species pairs, and whether or not coyote elicit the same behavioral response as something like puma or black bear across human development, while utilizing more of the data that Snapshot has to offer (only 82 of the nearly 4,000 sites across the total dataset were included), would make this publication more relevant to the general readership of PLoS ONE.**

*Thank you very much for the complimentary and thoughtful review of our manuscript. You raise an interesting point about additional species for analysis. We chose the three focal species for this study because they co-occur widely geographically across habitats and most importantly, all occur across a gradient of human development, which allowed us to test our hypothesis that human development alters the interactions between these species.*

*However, we agree that there is potentially more “meat on the bone” here. We have expanded the scope of our analyses by looking at additional species (more than doubling our candidate pool from 3 to 7). However, we felt that we still needed to be selective about the species we included because it was integral to testing our hypothesis that the selected species occur across the full range of development values. So – what we did was look at every species of meso and apex carnivore documented in the Snapshot USA program (N = 29 species of apex and mesopredator). We then evaluated the number of detections of each of these species occurring in each of the 3 categories of development (natural/low, medium, and high). We decided to include in our analyses all species that occurred in each of the 3 categories and were documented at a minimum of 5 Snapshot USA subprojects (sites) within each of the development categories so that we would be able to make statistical comparisons between the categories. This rubric allowed us to include an additional 4 species (for a total of 7) that increased the scope of our study and provided additional valuable insight into interspecific interactions. We thank you for this suggestion and hope that our approach is satisfactory.*

*We should also note that there is a misunderstanding regarding how much of the snapshot dataset we used. The review comments we received stated that we only used “82 of 4000 sites” and that’s not accurate. Snapshot USA has approximately 4000 cameras spread across ~120 study sites (subprojects in the terminology of Snapshot USA). We actually used a large number of the available subprojects (approximately 82 of 120). We only excluded those sites where our focal species (raccoons, coyotes, and opossum) did not co-occur such as in deserts and north of opossum range. While our new increased species pool necessarily allowed us to increase the number of sites we used, we have also tried to clarify this in both the methods and the first paragraph of results to show that we do indeed used/use most of the available snapshot data.*  **ABSTRACT
General Comments
The last sentence speaks to there being insufficient evidence that competitive or predatory pressures produced a behavioral response in the three species’ activity patterns. However, it says nothing about the relation to human development. As the change in activity in relation to human development was a central theme throughout the manuscript, it seems remiss to exclude any reference to it in the concluding marks of the abstract. As the information is currently presented, the relation to human development seems like an afterthought.

INTRODUCTION
General Comments
There is quite a bit of information in this introduction about predator-prey interactions. This seems completely unnecessary, as this is never studied herein and only alluded to once throughout. Furthermore, much of these references allude to the presence of apex predators on prey species, another point that is not hit on in this particular study. I either recommend: a) removing references to predator-prey interactions when unnecessary and replacing it with background information on co-occurrence between sympatric predators, or b) revamping the study like mentioned above to include apex predators and rewording the sections to discuss the impact they have on more subordinate carnivores.**

*In response, we have altered the introduction to shift away from mentions of the effects of apex predators on the activity of prey species and instead focus heavily on interspecific interactions and competition and how these factors might influence activity patterns. We have included several new references in support of these changes including:*

*Gallo T, Fidino M, Gerber B, Ahlers AA, Angstmann JL, Amaya M, Drake D, Gay D, Lehrer EW, Murray MH, Ryan TJ. Mammals adjust diel activity across gradients of urbanization. Elife. 2022 Mar 31;11:e74756.*

*Allen, M.L., Green, A.M. and Moll, R.J., 2022. Modelling the distribution and intraguild associations of an understudied mesocarnivore across the contiguous United States. Diversity and Distributions, 28(5), pp.1022-1033.*

*Hubbard, T., Cove, M.V., Green, A.M., Iannarilli, F., Allen, M.L., LaRose, S.H., Nagy, C., Compton, J.A. and Lafferty, D.J., 2022. Human presence drives bobcat interactions among the US carnivore guild. Biodiversity and Conservation, 31(11), pp.2607-2624.*

*Green, A.M., Barnick, K.A., Pendergast, M.E. and Şekercioğlu, Ç.H., 2022. Species differences in temporal response to urbanization alters predator-prey and human overlap in northern Utah. Global Ecology and Conservation, 36, p.e02127.*

*Frey, S., Fisher, J.T., Burton, A.C. and Volpe, J.P., 2017. Investigating animal activity patterns and temporal niche partitioning using camera‐trap data: Challenges and opportunities. Remote Sensing in Ecology and Conservation, 3(3), pp.123-132.*

*Gehrt, S.D., Anchor, C. and White, L.A., 2009. Home range and landscape use of coyotes in a metropolitan landscape: conflict or coexistence?. Journal of mammalogy, 90(5), pp.1045-1057.*

**Minor Comments
Lines 64-68: These statements need a references. Recent work (see Green et al., 2022 in Global Ecology and Conservation; Hubbard et al., 2022 in Biodiversity and Conservation; and Allen et al., 2022 in Global Ecology and Conservation) may better place them in the context of current work.**

*Thank you – we have incorporated these citations (here and elsewhere) to support the statements made.* **METHODS
General Comments
As discussed above, I feel like the authors should go deeper than they do, especially when utilizing a dataset as rich as the Snapshot USA one (incorporating data from less than 5% of sites seems like a disservice). There are many other mesocarnivores in the U.S. that occupy urban environments and have relationships with coyote and other mesocarnivores (e.g., see Allen et al., 2022 in Diversity in Distributions, which uses the entire Snapshot dataset). For example, it would be interesting to investigate the link between coyote and red fox, especially in relation to urban development, or the relationship between species such as skunk, raccoon, and red fox, as these species seemingly share very similar ecological niches. Furthermore, by looking at different assemblages of mesopredators across the U.S., the authors can leverage the full power of the Snapshot dataset.**

*Thank you for this suggestion. We have expanded our scope to include more species. However, we still limited this to species that occurred across the full range of human development so that we could still evaluate our hypothesis. See full justification above.*

**Lastly, descriptive statistics of the sites included in analysis, organized by strata, would be extremely beneficial. How many sites were there in each category? What was the spatial distribution of each category? What climates were included? What was the average precipitation and temperature across sites? Etc.**

*We have added language to clarify the stratification of sites used. This information can be found on lines 157-159. Details regarding how each site was classified were added into the methods as well, and can be found on lines 144-153. Classification is now based on the 2019 National Land Cover Database (NLCD; Dewitz, 2021).*

**Minor Comments
Lines 149-154: So Overlap coefficients were calculated on a site-by-site basis? In that case, was the error in site-specific estimates incorporated into the second stage of the analysis? It says that the site-specific confidence intervals were calculated, but I don’t see any reference as to how they were used or propagated though the second stage of modeling. I’m guessing the mean or median estimate from each site was used during the Kruskal-Wallis and Dunn tests. This information should be explicitly specified. Furthermore, if the inherent bias included in using an estimate from one model as an explanatory variable in another model (i.e., doing statistics on statistics) was not explicitly accounted for, than this needs to be mentioned as a caveat in the discussion or later in the methods.**

*We have added language to lines 163-167 to clarify this issue. We did indeed compare means across groups. We have deleted mention of bootstrapping which was incorrect and gave the false impression we were “doing statistics on statistics”.*

**RESULTS
General Comments
All four figures need some work. Specifically, both the x and y axes and the legend are too small, and there is a lot of unnecessary white space. It’s also pretty difficult to discern if any of the plotted differences are statistically significant. If possible, plotting the points as either different colors based on statical differences, or, more appropriately, using a letter designation above each comparison would help clarify this.**

*Thank you for the feedback and suggestions. Plots have been updated both to reflect the added species, but also to address comments here. Size of text along all axes were increased, and colors added for clarification (Fig. 2).*

**There needs to be a map of the study area provided, including sites that were included in analysis. With the current presentation, the reader has no idea where these data were gathered, other than it was across the U.S. A map would help contextualize the extent of these species’ ranges included in analysis, as well as the spatial configuration of sites.**

*Thank you for the suggestion – we have incorporated a map that shows these data. It is now* ***Fig 1****.*

**Minor Comments
157-158: Does this mean 82 sites per year or 82 sites across years?**

*Good question, this includes sites across the two years. With the updated species list, this now includes 210 snapshot sites, and clarifying language can be found on line 165 and 206-207.*  **157-159: What was the total sampling effort (i.e., total camera days across the 82 sites)?**

*Good question, we have added total sampling effort (trap nights, total detections, number of sites with detections and detections of each species to the results sections. This information can be found on lines 192-194.*  **DISCUSSION**

**General Comments**

**Typically, the first paragraph of the discussion section summarizes the main findings of the study and presents overarching takeaways that are then expanded on in further detail in the ensuing paragraphs. I particularly like this format, as I believe it is the easiest for readers to follow. Although it is not necessary, and is simply a matter of personal opinion, I urge the authors to consider reworking their first paragraph, at least.**

*Thank you for the suggestion, we have done this and included a summary paragraph.*

**Minor Comments**

**Lines 239-242: This line really reiterates the need for expanding this current study to incorporate the entirety of the Snapshot USA dataset. This is an extremely intriguing question, and identifying where and why species interactions occur or don’t occur would add great value to this work.**

**Lines 250-252: Again, this seems to signal rationale for this study to expand on this work and look large-scale with the intent of identifying why these differences occur.**

**Lines 252-255: Can we really say range wide here? Seems like quite a stretch.**

**Lines 258-260: This variation explained and summarized, either in a supplemental table or in the results section.**

**Lines 258- 269: I see no reason why this study cannot be the answer to the future research the authors call for.**

**Reviewer #2**

**I found this paper interesting, and I think it will make a good contribution to the Plos One journal. I do however have some comments (mostly minor) which I would like addressed. The majority of comments are attached in the document, but the main points I will highlight here;**

*Thank you very much for your positive review and helpful comments.*

**¬ There is a lack of information in this paper regarding the specific habitats and areas that camera data was collected. I think this is important to summarize, even if only into broad categories. Was the majority of data collected near towns? Agricultural areas? Forests? The use of the housing unit density index alone does not provide any of this info.**

*Thank you for the suggestion. We have added a table summarizing habitat for each site, as well as clarifying language. These can now be found on lines 160-162 and reference the table while introducing Snapshot USA on line 138.*

**¬ I am not convinced that the housing unit density was the best metric to test for temporal differences between species. I would like to see more argument why this was used over other metrics of human development.**

*You may be correct. We like the Housing Unit Density variable but it may not have been the best choice here. We have replaced it with an index of development derived from the National Land Cover Database which measures the impervious surface present in an area. This is more commonly used for these types of analyses and will be easier to understand for most readers.* **¬ Again, following on from the lack of habitat classifications, how common were agricultural areas in this study? In theory these would have low housing density, however meso-carnivores can have particularly high levels of human persecution in these areas, which could impact temporal activity, but not align with the low development idea. I would like to see some clarification on this.**

*Good question – we have added text to the methods and results and table x that show the breakdown of habitats. As you can see, agricultural areas are not much of a factor in this project.* **¬ I think it would be good for the authors to create a simple study map of the sites used in the analysis from Snapshot USA. I am curious to see the distribution of this data.**

*Great suggestion. We have added a map. It is now figure X.* **¬ The figures are blurry and need to be improved. I suggest also moving the axis titles further away from the axis.**

*Thank you for pointing this out. All figures have been fixed to reduce blurriness and are now publication quality.*
